# Research evolution in banking performance: a bibliometric analysis

**DOI:** 10.1186/s43093-021-00111-7

**Published:** 2021-12-14

**Authors:** S. M. Shamsul Alam, Mohammad Abdul Matin Chowdhury, Dzuljastri Bin Abdul Razak

**Affiliations:** Department of Finance, International Islamic University Malaysia, Jalan Gombak, 53100 Kuala Lumpur, Malaysia

**Keywords:** Banking performance, Banking efficiency, Bibliometric analysis, Web of Science, Biblioshiny, VOSviewer, Content analysis

## Abstract

Banking performance has been regarded as a crucial factor of economic growth. Banks collect deposits from surplus and provide loans to the investors that contribute to the total economic growth. Recent development in the banking industry is channelling the funds and participating in economic activities directly. Hence, academic researchers are gradually showing their concern on banking performance and its effect on economic growth. Therefore, this study aims to explore the academic researchers on this particular academic research article. By extracting data from the web of Science online database, this study employed the bibliometrix package (biblioshiny) in the ‘R’ and VOSviewer tool to conduct performance and science mapping analyses. A total of 1308 research documents were analysed, and 36 documents were critically reviewed. The findings exhibited a recent growth in academic publications. Three major themes are mainly identified, efficiency measurement, corporate governance effect and impact on economic growth. Besides, the content analysis represents the most common analysis techniques used in the past studies, namely DEA and GMM. The findings of this study will be beneficial to both bank managers and owners to gauge a better understanding of banking performance. Meanwhile, academic researchers and students may find the findings and suggestions to study in the banking area.

## Introduction

The financial services formed a significant contributory trademark in the overall economic growth by stimulating employment, offering vast avenues for investment and services to the consumers and the society [1]. Thus, economic development is led by economic growth whereby required capital is provided by the financial services [2]. Suggestively, capital creation by the financial services industry through accumulation and mobilisation of resources is considered the most crucial economic growth strategy component [3]. The banking system associates with creating funds by accumulating funds from surplus and channelling them to the investors as credit; those exhibit excellent ideas to generate a surplus in the economy but lack the capital to implement such ideas [4, 5]. Accordingly, the banking system plays a vital role to pledge the leading role of finance in economic development and promoting stable and healthy financial and economic development [6].

Banking performance has been regarded as a crucial factor of economic growth [7]. Efficiency and productivity change measures are rapidly used to evaluate banking performance. Academic researchers have been focusing on the efficiency and productivity of banking institutions for a long period, while economic growth is carried out in the discussions. Discovering research activities on banking efficiency and productivity in economic growth enables researchers to identify the local and international input to this particular discipline. More so, it will enable researchers to identify the ‘hot spots’ discussed by academic researchers and find the research gaps [8]. Indeed, banking performance in standings is a broad scientific topic, and estimating research activities might not be useful. For instance, research activities in this area extended to several constituents such as methodological approaches, banking approaches. In the current study, banking efficiency and productivity are considered as banking performance that contributes to the economic growth of an economy. Therefore, the main objective of this study is to explore the research activities of banking performance to economic growth. The investigation of banking performance research activities will enable the researchers to find the present directions of the research area and thus speculates the future research suggestions. Besides, it will also enable to expound the depth of past research activities and themes on banking performance relating to the economic growth measurements.

The use of the bibliometric method is appropriate to demonstrate the research shape and activity, volume and growth in a specific discipline [9]. A bibliometric method is a quantitative application of bibliometric data [10]. It analyses a wide-ranging quantity of published research articles employing the statistical tool to identify trends and citations or/and co-citations of a certain theme by year, author, country, journal, theory, method, and research constituent [11]. Significantly, this technique further distinguishes key research themes and active researchers, countries and institutions for future research planning and funding [12]. Scholars apply this method for several reasons: to reveal emerging trends in published research articles and journal performance, cooperation patterns, and research elements, and to reconnoitre the intellectual edifice of an exact domain in the existing literature [9, 13].

Minimal studies have used bibliometric analysis related to banks. For instance, Violeta and Gordana have employed bibliometric analysis to spot the trends of DEA application in banking [14]. Another study conducted by Ikra et al. applied the bibliometric method to Islamic banking efficiency [15]. By an extensive search on the Scopus, Web of Science and Google Scholar, no such study was found related to bibliometric analysis on banking performance to the economic growth. Nevertheless, this study will be the first attempt to conduct bibliometric methods on the banking performance to the economic growth that could be the basis for future studies.

The findings of this study unfolded several contributions to both policymakers, bank managers and academic researchers. Firstly, the findings would benefit the policymakers regarding the contribution and trends of banking performance. It would allow them to take necessary initiatives to promote and improve banking performance, thus economic development. Meanwhile, bank managers may utilise the findings to strengthen their banking operations by acknowledging key factors that contributed to the performance. Finally, academic researchers are enabled to detect the current trend and topics related to the banking area that leads to further studies.

## Methods

Bibliometric analysis has achieved enormous popularity in social sciences research in the current years [9, 16–18]. The popularity of bibliometric analysis is observed from the development, accessibility and availability of software, for instance, Leximancer, Gephi, VOSviewer, Biblioshiny and publication databases (Web of Science and Scopus). Further, the rapid growth of bibliometric analysis in scientific production has emerged from business research to information science [9]. The popularity of bibliometric methodology in social science research is not a trend but moderately an image of its usefulness for constructing high research impact by handling excessive scientific data [9].

The bibliometric analysis is beneficial for briefing the trends in the research documents classifying ‘blind spots’ and ‘hot spots’, and finding a more inclusive understanding of the published research documents [19]. In detail, this analysis empowers the recognition of the most advanced (hot spots) and the less established topics (blind spots) within the documents that, shared with other bibliometric procedures, recommend future research avenues. The bibliometric analysis uncovers several ascriptions, such as unveiling emerging trends in documents and the performance of journals, research constituents and collaboration patterns and discovering the intellectual edifice of an exact domain in the existing literature [13, 18]. The data that apply in this analysis incline to be immense (hundreds, thousands) and unbiased in nature (publications and citations number, keywords occurrences and topics). However, its explanations often depend on both subjective (thematic analysis) and objective (performance analysis) assessments formed through well-versed techniques and procedures [9]. Therefore, this study applied bibliometric analysis to examine the general perspective on banking performance and economic growth.

Two categories are mainly manifest in the bibliometric techniques, namely, performance and science mapping. Precisely, research elements’ contributions are accounted for in the performance analysis, while the connections between research elements are focused on science mapping [9]. This study follows performance analysis, science mapping and network analysis suggested by Donthu et al. [9].

### Data extraction process

Two primary databases, the Web of Science and the Scopus, are commonly used in the bibliometric analysis [20]. Both databases are prominent for the peer-reviewed published research articles. The data for this analysis were a collection of bibliographic data from the Web of Science. The Web of Science (WoS) is a multidisciplinary online database providing access to several citation databases, namely Science Citation Index Expanded (SCIE), Social Sciences Citation Index (SSCI), Emerging Sources Citation Index (ESCI), Arts and Humanities Citation Index (AHCI), Conference Proceedings Citation Index, Index Chemicus and Current Chemical Reactions [18, 21].

**Fig. 1:**
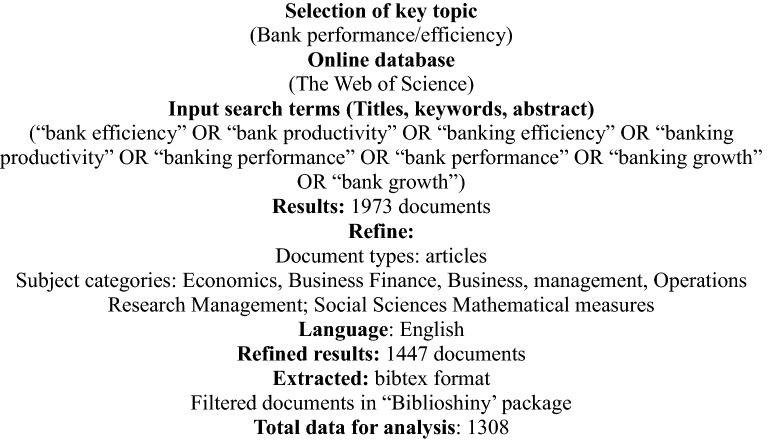
Data extraction process

This study has applied a two-stage data extraction process, following Bretas and Alon, Alon et al. and Apriliyanti and Alon [16, 22, 23] as shown in Fig. 1. The choice of the keywords is crucial to ensure that it covers the total body of published documents on banking performance and economic growth [21]. Accordingly, the selection of keywords was carried out by reviewing several abstracts and authors’ keywords in most related literature on the Web of Science. The selected keywords were executed in the WoS online database on 9 August 2021. A combination of keyword search terms was considered; (1) ‘banking performance*’ to nail all discrepancies of the term such as the role of the bank, bank efficiency, bank productivity, banking efficiency, banking productivity, banking performance, bank performance, upon refining the search by including only research articles from the categories; economics, business finance, business, management, operations research management, social sciences mathematical measures and documents written in English.

The second stage extracted raw data from the online database combined, checked for duplicate documents and merged using ‘R’. Further, the documents were filtered in the ‘biblioshiny’ tool to omit book chapters and conference proceedings. After the extraction process for the bibliometric analysis, several impactful documents were selected based on local and global citations to conduct content analysis. The content analysis allowed the researcher to identify the leading research scopes and trends. Further, it allows identifying the streams and recommendations for future studies [22]. A total of 36 documents were selected to conduct a comprehensive review and valuation of the documents.

## Results

### Performance analysis

Performance analysis investigates the contributions of academic research elements to a particular discipline [24]. This analysis is naturally descriptive, which is the hallmark of bibliometric analysis [9]. It is a standard method in reviews to exhibit the performance of various research elements such as authors, countries, institutions and sources similar to the profile or background of respondents generally presented in empirical studies, albeit more statistically [9, 18]. Many measures exist in the performance analysis; hence, the most protuberant measurements are publications number and citations per research constituent or year. The publication is considered productivity, whereby citation measures influence an impact [9]. Besides, citation per document and *h*-index associate both publications and citations with evaluating research performance [18].

Table 1 presents the publication’s performance of banking performance. The results show a total number of 1308 documents published from 1972 to the present. Among 2275 contributed authors, a total of 202 authors were solely, and 2106 authors collaborated to the publications. A total of 31,458 citations received by published documents lead to an average of 629.16 citations per year, while 775 in *h*-index and 1023 in *g*-index. Hence, the banking efficiency field acknowledged productivity of research published by an average of 26.16 documents per year whereby nearly two authors (CI = 1.9) published one article, and standardised collaboration is 0.43 (between 0 and 1).

**Table 1:** Metrics for performance analysis

Metric	Description	Result
Total publications (TP)	Number of total publications	1308
Number of contributing authors (NCA)	Total of number of contributed authors	2275
Single-authored documents (SA)	Number of single-authored publications	202
Co-authored documents (CA)	Number of co-authored publications	2106
Number of active years of publication (NAY)	Total periods of publications by research area	50 years
Productivity per active year of publication (PAY)	Total publications/number of active years of publication (TP/NAY)	26.16
Total citations (TC)	Total citations received by published articles	31,458
Average citations (AC)/year	Average citations per year of publications	629.16
Collaboration index (CI)	The extent of collaboration {(NCA/TP)/TP}	1.9
Collaboration coefficient (CC)	Standardises the extent of researcher collaboration between 0 and 1 {1-(TP/NCA)}	0.43
*h*-index (*h*)	*h* Number of documents cited at least *h* times (a measure of influence)	775
*g-*index *(g)*	*g* Number of documents cited at least *g*^2^ times (a measure of impact)	1023

**Fig. 2:**
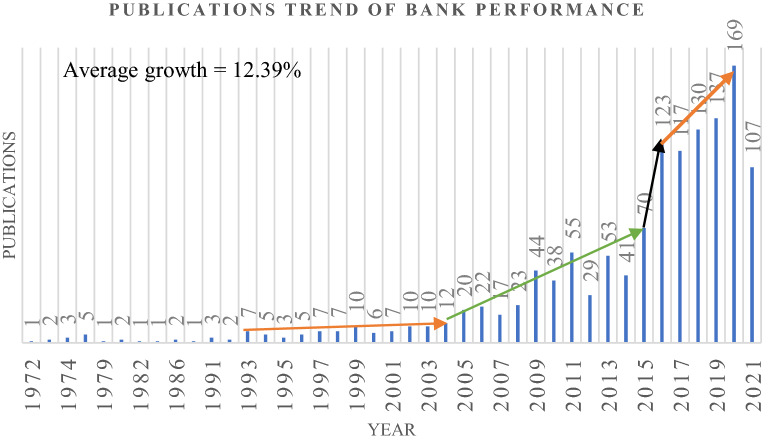
Annual Scientific production

The annual production of scientific publications on banking efficiency is presented in Fig. 2. The first research article related to banking performance was published by Fraser and Rose [25], who studied the effect of new bank appearance in the market on bank performance. The annual growth of publications on banking performance or banking efficiency is recorded to 12.39%. The publications are significantly increasing in recent periods, especially from 2016 to the present. However, the mandated growth in publications is observed between 2004 and 2015, while earlier periods (1972–2003) were quite sluggish. In these consequences, academic researchers have started to focus on banking performance or banking efficiency in the recent period. As a result, it can be concluded that banking performance and its sphere are shaping upwards through the research contributions.

### Science mapping

Science mapping investigates the connections between research elements [26] that relates to the intellectual connections and structural networks within research constituents [9]. The science mapping includes citation analysis, bibliographic coupling, co-citation analysis, co-occurrence network, collaboration techniques. When combined with network analysis, these techniques are instrumental in exhibiting the research area’s bibliometric edifice and intellectual structure [27].

### Citation analysis

The citation analysis is a fundamental approach for science mapping that runs on the assumption that citations reproduce intellectual contributions and impact the research horizons [28]. This analysis shows the impact of published documents by measuring the number of citations they received [9]. Accordingly, it enables the discovery of the most influential and informative documents in a research constituent. Thus, it allows gathering insights into that constituent’s intellectual dynamics [9]. Table 2 presents the top 20 impactful and influential documents in the field of banking performance or efficiency. The analysis has discovered that a total of 1112 documents (85%) out of 1308 documents received global citations. The global citations refer to the number of citations received in the overall Web of Science citations. However, 196 documents (about 15%) have not received any citation; meanwhile, 130 documents (about 10%) received only one citation. A document written by Berger An received the highest number (665) of citations which was published in 1997. The second most influential document was written by Seiford [51] received a total of 549 citations, followed by the document written by Back (2013) received 512 citations. In fact, a total of four documents written by Berger An rank in the top 20 impactful research articles in the field of banking performance or efficiency.

**Table 2:** Top 20 most cited papers. *Source*: Biblioshiny R package

Document	Total Citations	TC per Year
Berger An (1997), J Bank Financ	665	26.60
Seiford Lm (1999), Manage Scienc	549	23.87
Beck T (2013), J Bank Finance	512	56.89
Beltratti A (2012), J Financ Econ	484	48.40
Berger An (2013), J Financ Econ	463	51.44
Bonin Jp (2005), J Bank Financ	451	26.53
Berger An (2009), J Bank Financ	438	33.69
Fahlenbrach R (2011), J Financ Econ	390	35.45
De Andres P (2008), J Bank Financ	359	25.64
Aebi V (2012), J Bank Financ	303	30.30
Mester Lj (1996), J Bank Financ	286	11.00
Ariss Rt (2010), J Bank Financ	274	22.83
Beck T (2010), J Financ	272	22.67
Micco A (2007), J Bank Financ	268	17.87
Hermes N (2011) World Dev	263	23.91
Berger An (1993), J Bank Financ	253	8.72
Fiordelisi F (2011), J Bank Financ	252	22.91
Casu B (2003), Appl Econ	236	12.42
Garcia-Herrero A (2009), J Bank Financ	228	17.54
Lin X (2009), J Bank Financ	225	17.31

Factually, the majority of the documents without citations was published in a recent period. At the same time, the highly cited documents were published quite earlier. To detect the immediate influence of more recent documents is to apply the measurement of an average citation per year [29]. By evaluating the average citations per year, nine out of ten documents are also among the top 10 documents. Perpetually, Beck [45] holds the highest number of average citations per year (56.89), followed by Berger An (2013) ranked second position (51.44) and Beltratti A (2012) ranked the following position (48.40). Based on the citation analysis, it can be elucidated that Berger An is the most influential author in the banking efficiency research constituent.

### Co-occurrence analysis

**Fig. 3:**
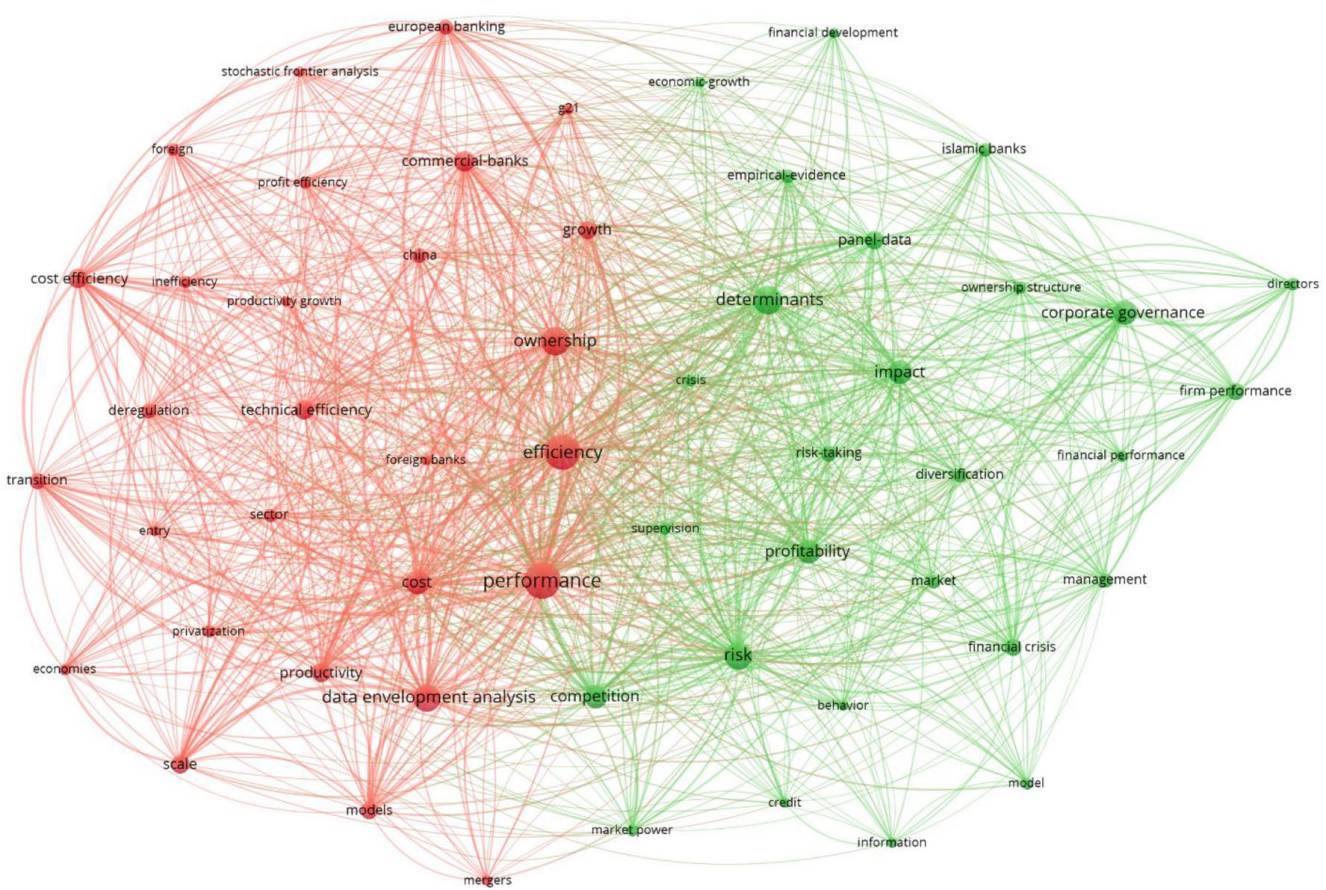
Co-occurrence of keywords, Tool: VOSviewer. *Note* the nodes represent the keywords, and the edges between words present their occurrence of interactions. Each colour of nodes represents a cluster/theme. The size of the node presents a greater frequency of occurrence

Co-occurrence analysis was projected by Callon et al. [30], considered as content analysis that is useful in plotting the strength of connotation within keywords in textual data. In other words, co-occurrence analysis is an approach that investigates the actual content of the document itself [9]. It maps the pertinent literature straight from the associations of keywords shared by research articles [24, 27, 31, 32]. The co-occurrence analysis deduces words to appear recurrently in a cluster. It exhibits conceptual or semantic groups of various topics or sub-topics considered by research constituents [9, 24]. Cobo and Herrera signified that spotted clusters could be applied with few objectives [24]. For instance, they can be applied to analyse their progression by gauging extension across successive subperiods and measuring the research area through performance analysis. Figure 3 displays the co-occurrence of keywords within the bank efficiency research constituent. As the focus of this research, bank performance represents the larger node associated with corporate governance, financial performance, financial crisis, nonperforming loans and others. In these scenarios, the red-coloured cluster depicts that these subtopics or variables are directly associated related to the bank performance theme due to repetitive co-occurrence of those words. Likewise, the green-coloured cluster represents a theme related to bank efficiency associated with performance and ownership. In the same cluster, the nonparametric data envelopment analysis is extensively used to measure commercial banks' technical and cost efficiency and productivity. Parametric stochastic frontier analysis is narrowly observed in efficiency measurements comparably. The green-coloured cluster depicts the determinants of bank profitability including other impactful variables such as risk, competition, corporate governance. This cluster applied panel data in order to examine performance, financial development as well as economic growth. Each of the cluster identifies the interacted themes used in the published documents using co-occurrence of keywords.

### Collaboration networks

**Fig. 4:**
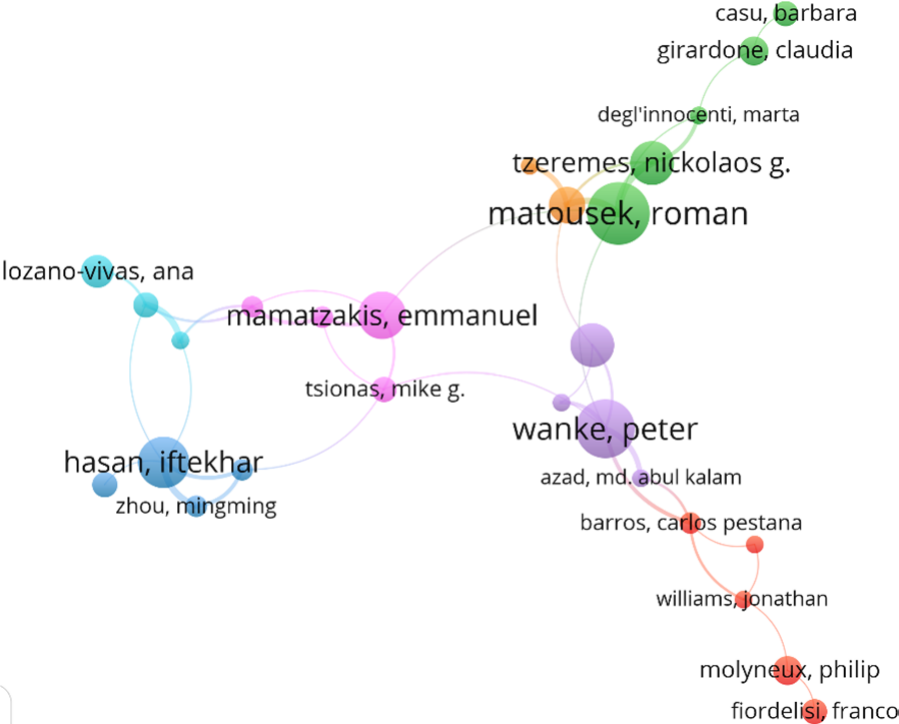
Authors’ collaboration networks. *Source*: VOSviewer. *Note* the nodes represent the authors, and the size represents the frequency of contribution, the colour presents a cluster or a particular group, and the link shows the link among authors that collaborated for research articles

Collaboration analysis explores the associations within researchers in a particular constituent. It is a formal way of intellectual association among researchers [33, 34]. Therefore, it is crucial to understand how researchers associate among themselves [9]. In the presence of growing theoretical and methodological complexity in research, intellectual networking (collaboration) has become commonplace [33]. Indeed, collaboration or interaction among researchers enables improvements in academic research; for instance, greater interactions among diverse researchers allow richer insights and greater clarity [35]. Researchers who collaborate form a network named ‘invisible collages’ whose research can help improve undertakings in the study field [36]. Figure 4 presents the collaboration network of authors those co-authored academic articles in banking efficiency. Based on the collaboration network, Wanke P (Universidade Federal do Rio de Janeiro) was the most collaborated author who co-authored with four authors from different institutions in different countries. At the same time, Matousek, R (University Kent), Hasan, I (Rensselaer Polytechnic Institute) and Mamatzakis, E (University of Sussex), have also exhibited as greater collaborative researchers. In these consequences, authors from different institutions and from different parts of the world are collaborating to the banking performance/efficiency field.

### Bibliographic coupling

**Fig. 5:**
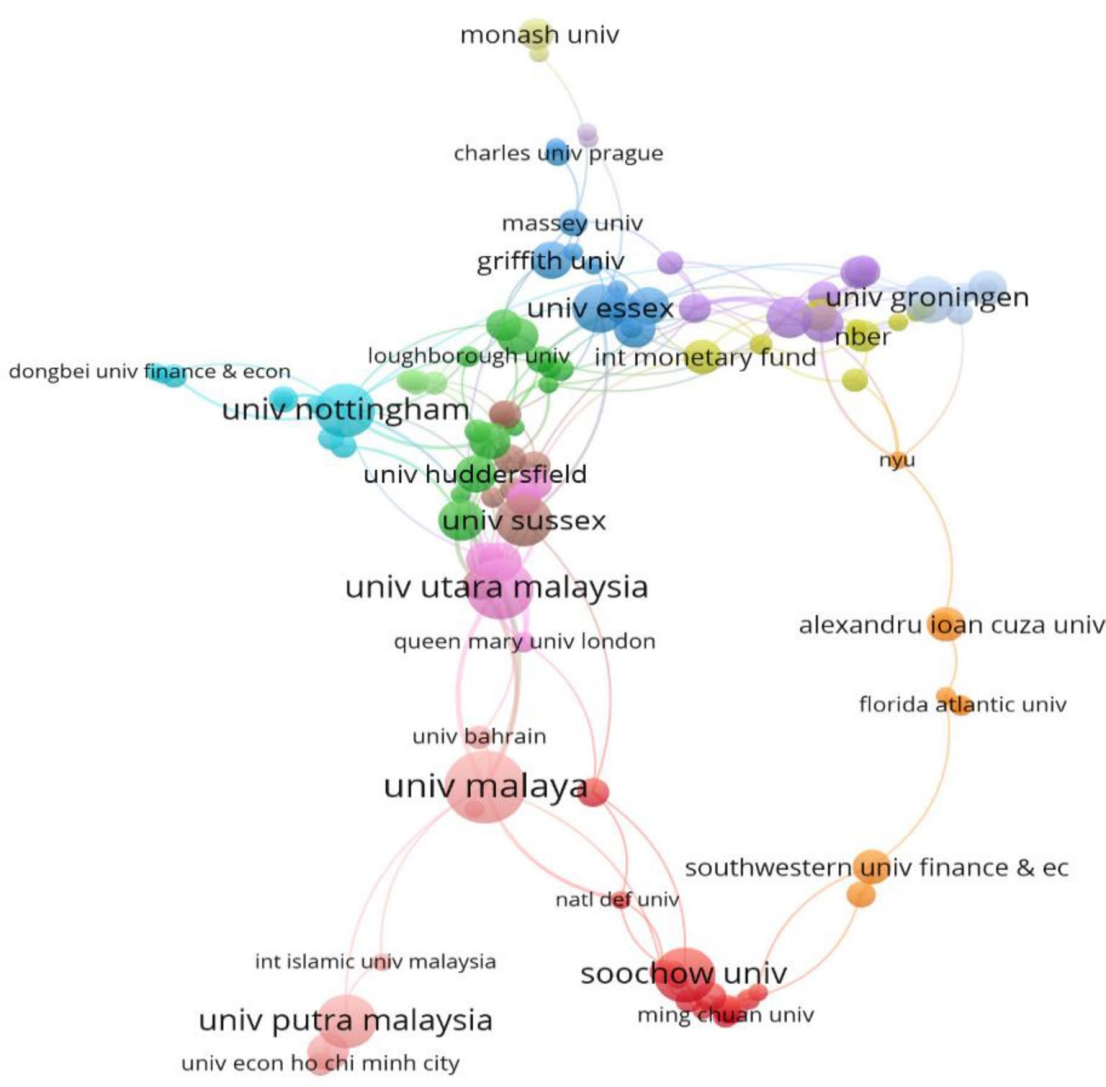
Bibliographic coupling of author-affiliated institutions. *Source*: VOSviewer

Co-authorship or collaborative networks within the authors and other crucial facets in the collaboration networks are the collaboration of author-affiliated countries and institutions [31]. Figure 5 exhibits the collaboration network within authors’ affiliated organisations. University Malaya and University Utara Malaysia, University Malaya and University Putra Malaysia, University Malaya and University Fed Rio de Janeiro all depict a strong collaboration network. In general, all the institutions display an embellishment among the institutions within the same region.

**Fig. 6:**
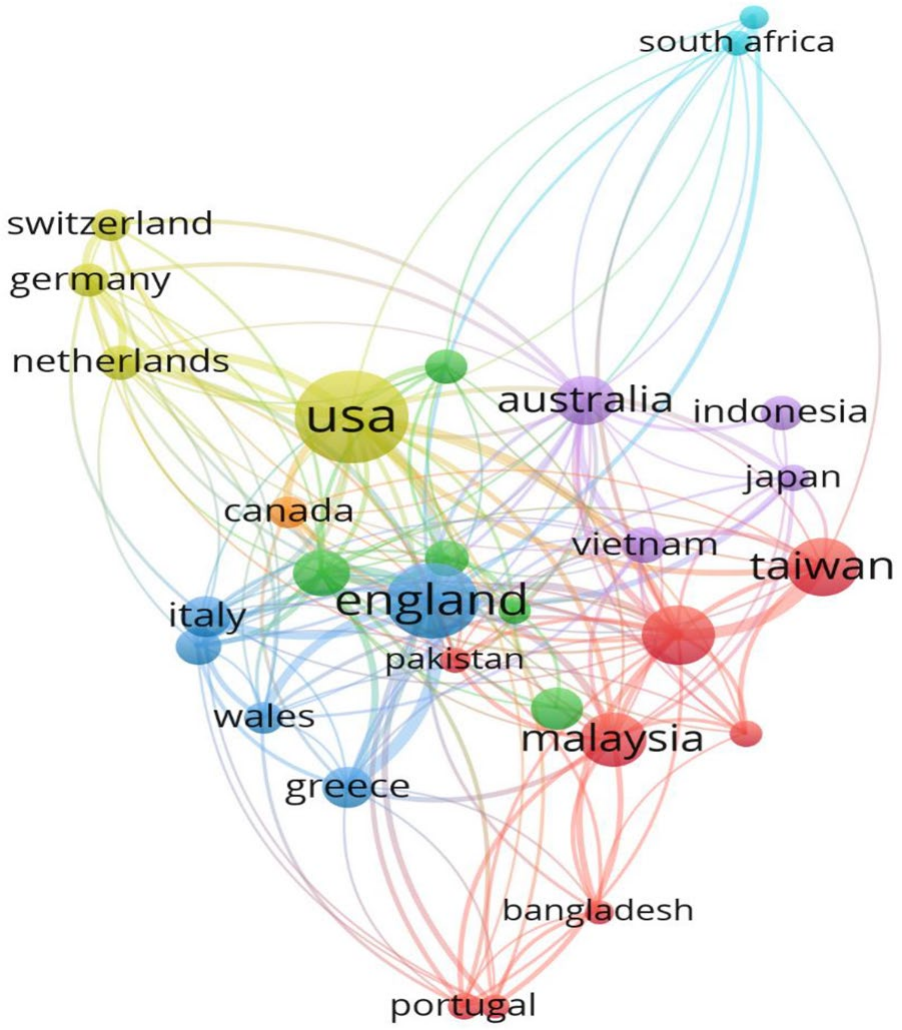
Collaborative authors’ affiliated countries

Similar to co-authors’ affiliated institutions, the collaboration of authors’ country presents a steady association among authors’ connections that allow exploring comparative and concurrent research works. Figure 6 represents the network of collaborative authors’ affiliation countries. These countries include South Africa and the USA, England and the USA, Australia and the USA, Malaysia and the USA, Germany and the USA, representing a high proportion of authors’ affiliated institutions are in the USA with this country performing as a hub of co-authorship publications from 1972 to 2021.

## Discussion

This study discusses trending themes based on the bibliometric findings and reviews of highly cited and most recent documents (see Appendix 1). It also indicated the type of study, theories, methods and main findings to suggest comprehensive future studies.

## Research directions

**Fig. 7:**
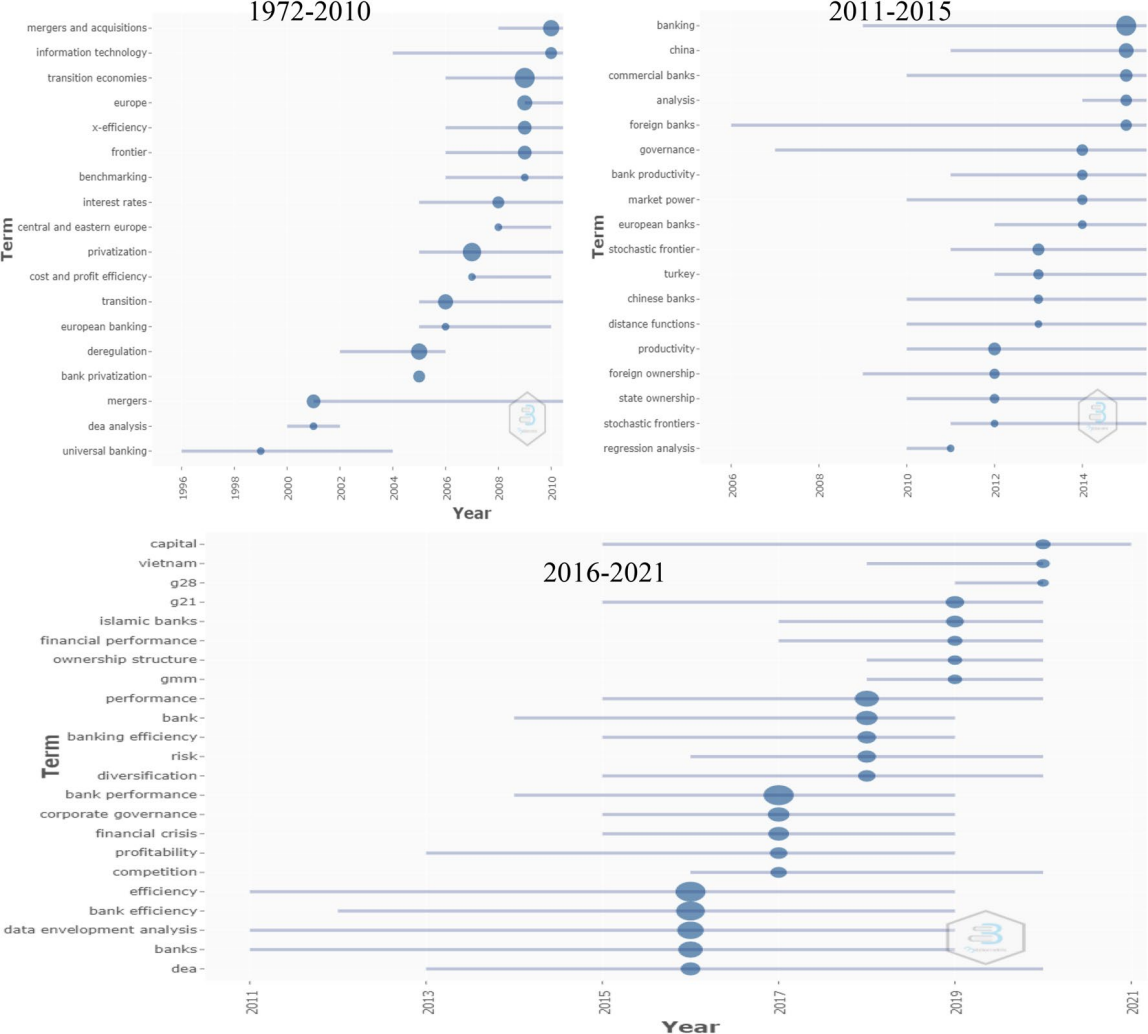
Trend topics in different periods. *Source*: Biblioshiny analysis. *Note* the frequency of terms selected 3 times for 1972–2010, 5 times for 2011–2015, 10 times for 2016–2021

Between 1991 and 2010, studies related to banking performance have posited several antecedents to banking performance. Figure 7 displays the trend topics based on author keywords that appeared between 1972 and 2010. Studies in this period mainly focused on mergers and acquisitions, information technology and transition economies that emerged from universal banking deregulation and bank privatisation. The financial crisis during 2008–2009 drew the attention of scholars to evaluate the banking performance. Idiosyncratically, this phenomenon has been acknowledged by researchers from 2010 to 2015, focusing on the role of corporate governance in the performance of the banking industry, including compensation, risk management, determinants of stock returns, capital buffer, productivity. Idiosyncratically, a vast of studies were conducted on Chinese commercial banks and the effect on their economic growth.

In the recent period (2016–2021), diverse factors posited in the studies that dominantly present a significant interest from banking scholars. While studies earlier mainly focusing on efficiency and its contributing factors, recent periods extended research directions to multiple constituents. For example, how banks diversified their services and the role of human capital efficiency to the banking performance [37]. Bose et al. employed the effect of green banking on the performance that underpins the inclusion of the environmental sustainability approach by the banking industry [38]. Meanwhile, Bhattacharyya et al. showed the effect of CSR expenditures and financial inclusion on the performance that define the social sustainability indicator of the banks [39]. Repeatedly, the role and structure of the board, categorisation of deposits and loans, risk exposures (business cycle), macroeconomic factors were also acknowledged in recent banking performance studies [40–43]. Idiosyncratically, scholars recently focus the components of sustainability of the banking industry from economic, environmental and social aspects [44]. Furthermore, the effect of banking and its stability on economic growth has been broadly carried out in the recent period. Moreover, the development of studies was taken into account, which implies the contribution to the economic growth of particular regions. Based on the earlier and recent studies, it is precisely observed the diversification of research constituents in relation to bank performance studies. Earlier studies (up to 2015) mainly measured banking performance or efficiency based on accounting measurements, while recent studies started to include market measurements principally based on stock returns performance. On the other hand, the rise of Islamic banking and finance influenced academic researchers to compare the business models [45], banking efficiencies [46] between conventional and Islamic banks, and efficiency for Islamic banks [5].

Based on the review of impactful documents published from 1990 to 2010, two particular objectives were identified: the effect of the board of directors or ownership on the bank performance [47–49] and measurement of efficiency, including cost and profit efficiency [50–52]. These constituents extended during 2011–2020 by the inclusion of risk-taking management [53], CEO incentives [54], contributing factors including capital, banking crises on banking performance [42, 55–57]. Meanwhile, the Islamic banking system got crucial attention from academic researchers. Accordingly, several studies evaluated and compared efficiency between Islamic and conventional banks [45, 58, 59]. Nevertheless, the role of the banking industry in economic growth was included in the research constituents in the recent decade. For example, Xu, Santana and a few more scholars investigated the correlation between financial intermediation and economic growth [57, 60, 61]. In recent years, scholars extended the banking-related research constituents to diverse areas. The effect of human capital efficiency [37], green banking [38], CSR expenditures [39] and bank stability was included to measure banking performance. These extensions of research themes within banking performance studies posited a significant interest by academic researchers.

Apparently, almost all documents adopted the quantitative method in measuring banking performance research constituents. However, studies that measured banking efficiency mainly applied nonparametric analysis DEA [5, 51], while SFA was adopted by limited studies [37, 42, 43]. On the other hand, regression analysis was predominantly applied to investigate banking performance from 1990 to 2010 [49, 50]. In recent studies, academic researchers have vastly adopted GMM (generalised method of moments) to examine the contributing factors on banking performance [39, 42, 57, 60]. These methods are dominating the banking-related studies throughout the publication periods. Over the periods, scholars have developed DEA applications in several categories, such as bootstrap, networking. Meanwhile, GMM with different approach (dynamic and system) techniques exploited panel data primarily extracted from Bankscope, Datastream, annual reports etc.

### Main findings

Earlier, banking inefficiencies were substantially observed low, negatively affecting profitability and marketability [50, 51]. This trend was continuously depicted in studies [52]. However, Berger et al. evidenced better efficiency for larger banks than smaller banks [50]. On the contrary, Seiford and Zhu posited an adverse effect of bank size on marketability [51]. More so, Rehman et al. found larger banks are less efficient than smaller banks [40]. Hence, Moudud-Ul-Huq posited diverse impacts of bank size and competition on performance [62]. So, banking size is deemed to have a substantial effect on the overall performance of banks. However, Adesina embellished that diversification of services and choices of management decisions on loans (nonperforming, debt issuances) [63, 64] and deposits [41] affect the banking performance [37]. Moreover, board structure affects banking performance [40, 65], while higher human capital efficiency enhances banking performance [37].

Generally, foreign-owned banks provide better service, greater profitability and are better efficient than local banks. This phenomenon was evidenced in several studies; for example, Bonin et al. and other scholars demonstrated that foreign-owned banks are more cost-efficient than other banks [48]. However, this trend did not exist for Islamic banks as local banks showed better efficiency than foreign peers [58] and more efficient than conventional [59]. Meanwhile, state-owned or government-owned commercial banks were less efficient and provided poorer services [48, 49, 52]. But these banks’ efficiency was higher than urban/rural banks during credit risk shock [41]. Nevertheless, banking efficiency and performance substantially depend on diversification of services, managerial adequacy, ownership, types and size.

Studies have evidenced financial development and thus the banking industry’s role in economic growth [60]. In the nineteenth century, the establishment of the savings bank demonstrated city growth in Prussia [66]. Potentially, banks provide investment capital to increase per capita GDP [43]. However, Haini documented a contrasting effect of banking development on economic growth through a push out of private investment due to high levels of the banking sector [67]. However, Stewart and Chowdhury proved that a stable banking sector lessens the negative impact of a crisis on GDP growth and provides economic resilience in both developed and developing countries. Overall, findings elaborated a crucial link between banking sector development and economic growth.

### Future study suggestions

This study has recommended several scopes for future studies in the hybrid review, mainly through bibliometric findings and the structured review of impactful articles [11]. In other words, the recommendations for future studies are made by observing and analysing discussions on highly cited and recent cited documents. Overall findings and analyses raised several questions that need to be addressed for future studies.

Firstly, does the banking sector improve economic growth in the least developed countries? Prior studies mainly focused on developed and developing economies, but less attention was given to least developed countries. Secondly, vast studies investigated contributing factors of banking performance, while political instability has been ignored. Future studies might include political instability on the banking performance. Apart from it, nonperforming loans can be another addition to future studies, and even few studies documented it. Thirdly, how do banks perform during the pandemic crisis, for instance, COVID-19? The current pandemic crisis can be a significant factor in banking performance related to future studies, including efficiency, mortgages, loan recovery, deposits and business services. The studies can include consumer behaviour (due to restricted movements, safety measurements), green banking (online transaction and services), financial technologies (inclusion of nonbanking services) and the contribution or continuance of economic activities in the country during and after the pandemic crisis.

Significantly, prior studies have ignored the current trend of FinTech inclusion in banking performance. Fourthly, will FinTech takeover the banking services and diminish banks in the near future? Future studies may investigate the effect of FinTech applications on banking. More so, future studies may explore the banking industry’s barriers, challenges and threats due to FinTech growth. Fifthly, almost all studies employed quantitative analysis related to banking performance. Therefore, future studies may use qualitative methods to explore the opportunities and practices of banks and their performance. Sixthly, the majority of the studies either applied parametric or econometric techniques to investigate the bank performance. Recent developments in technologies and methods may provide easy and robust results in such related studies as using machine learning for data analysis and predicting banking efficiency and productivity determinants. Seventhly, past studies mostly followed the intermediation approach, which scarcely included production and operating approach measurement. Future studies may extend the efficiency analysis using productivity growth analysis. Further, the majority of the studies observed efficiency only. Future studies can include a productivity change index along with an efficiency analysis. Finally, GMM and regression were broadly applied to investigate the effect of antecedents of banking performance and link to economic growth. Future studies may adopt other advanced data analysis techniques such as partial least squares, structural equations and other econometric techniques.

## Conclusions

The main purpose of this study is to explore the trends and research activities in banking performance and the economic growth research domain. To achieve this objective, a bibliometric analysis was applied and performed several analyses, namely citation, co-occurrence of keywords, the collaboration between authors and coupling between institutions and countries, and discussion by reviewing most cited and most recent influential research articles. This study presents the most common themes, sub-themes associated with highly cited documents and authors; furthermore, the content analysis identified the research directions, research objectives, methodologies, topics and findings.

Based on the reviewing literature, the efficiency theory, banking theory mainly intermediation approach and nonparametric technique, namely data envelopment analysis along with econometric method, regression was used in the published documents. The findings of this study, along with future study suggestions, could be beneficial to bankers as well as academic researchers and students studying banking performance and its role in the economy.

## Limitations

The most crucial limitation in any bibliometric analysis is the database selection. It means selecting the data and the limits of its interpretation [68]. This study has three key limitations; firstly, it has chosen ‘Web of Science’, one of the largest online databases to gather data on banking performance research articles from 1972 to 2021 and refined based on subject categories and language (English). The database could be improved if other databases were included and also if book chapters and conference proceedings were added. Secondly, the selection of keywords; although selected keywords are deemed to be most relevant to encompass the majority of articles related to banking performance, there is always an opportunity to search further articles by using additional keywords. Lastly, this study could not conduct co-citation analysis due to the unavailability of cited documents in Web of Science data format.

## Data Availability

The data collected from the Web of Science online database were saved on Microsoft excel and remained with authors. The data are available upon request.

## Appendix 1: Reviewed documents

**Table:**

Authors	Type of paper	Objective	Theories/approach	Methods (sample & technique)	Main findings
Berger et al., [50]	Quantitative	Derived profit function model and measured inefficiency		Multiple regression, annual reports	US banks’ inefficiencies were quite large, while almost half of the potential profit variables were lost to inefficiency. Further, larger banks were substantially efficient than smaller banks
Berger and DeYoung [63]	Quantitative	Investigated the intersection between problem loan and bank efficiency		Granger-causality (OLS, GLS), Panel data	Problem loans and measured cost efficiency posited unidirectional links; problem loans (nonperforming loans) precede reductions in cost efficiency, and cost efficiency precedes a reduction in problem loans
Seiford and Zhu [51]	Quantitative	Evaluated the performance of the top 55 US commercial banks		Output-oriented (CCR) DEA, (BCC) DEA, Annual reports	90% of US banks were found inefficient relating to profitability and marketability. Besides, bank size was suggested to have a negative effect on marketability
Bonin et al. [49]	Quantitative	Examined the effects of ownership on bank efficiency		Regression (stochastic frontier estimation), panel data	Foreign-owned banks are more cost-efficient than other banks and provide better service, while government-owned banks are less efficient in providing services, and better banks were privatised first in transition countries
Micco et al. [48]	Quantitative	Assessed the link between ownership and bank performance		Regression, panel data	State-owned banks had lower profitability and higher costs than private banks in developing countries, while foreign-owned banks showed the opposite. Further, political considerations, especially during election years, differed performance between public and private banks
Andres and Vallelado [47]	Quantitative	Analysed the effectiveness of the board directors in the bank		Regression (OLS, system estimator regression), Panel data	An inverted nonlinear relationship was found between bank performance and board size, between the proportion of nonexecutive directors and performance. However, bank ownership, intuitional differences and weight of the banking industry differ in the relationship
Berger et al. [52]	Quantitative	Measured cost and profit efficiency		Regression, Annual report	Big Chinese banks are inefficient, while foreign banks were the most efficient
Fahlenbrach and Stulz [54]	Quantitative	Investigated the effect of CEO incentives on bank performance during the crisis		Regression, S&P,	Banks with higher CEOs’ incentives performed worse while did not perform worse during the financial crisis. CEOs had lost extremely large wealth as they did not reduce their shareholdings during the crisis
Aebi et al. [53]	Quantitative	Examined the effect of risk management related corporate governance mechanisms on bank performance during the financial crisis		Regression, Panel data	Banks’ stock returns and ROE exhibited higher for those credit risk officers directly reported to the board directors rather than CEO during the crisis
Beltratti and Stulz [55]	Quantitative	Measured the contributing factors to the poor bank performance during the credit crisis		Regression, Panel data	The fragility of banks financed with short term capital market funding and the better performing banks had less leverage and lower returns immediate before the crisis
Berger and Bouwman [56]	Quantitative	Investigated the effect of capital on bank performance	The screening-based theory, The asset-substitution moral hazard theories	Logit survival regressions, OLS	The capital was found to enhance the survival chances and market shares of small banks always even during bank crises, market crises, and normal periods. Large and medium-sized banks were linked by capital predominantly during banking crises and the ones with limited government intervention
Beck et al. [45]	Quantitative	Compared efficiency between conventional and Islamic banks		Regression	Islamic banks had greater intermediation ratios, higher asset quality and better capitalisation over conventional though they were less efficient. hence, Islamic banks performed better during financial crisis regards of asset quality and capitalisation
Xu [60]	Quantitative	Investigated the relationship between financial intermediation and economic growth		System GMM, dynamic panel data	A diverse measure of financial development was generally linked to economic growth. The size and depth of the financial sector significantly influence economic growth
Kamarudin et al. [58]	Quantitative	Examined and compared the efficiency of domestic and foreign Islamic banks in SEA countries		DEA, Annual Reports	Domestic Islamic had greater efficiency than foreign banks peer
Moudud-Ul-Huq [42]	Quantitative	Investigated the linkage between capital buffer, risk and efficiency adjustments		SFA, GMM, panel data	The economic cycle had a substantial effect on capital holding, risk and efficiency adjustments. Besides, high capitalised banks posited less efficient than low capitalised banks due to enact of regulatory pressure
Buallay et al. [44]	Quantitative	Examined the relationship between sustainability reporting and bank performance after financial crisis	Value creation theory	Regression, GMM, panel data	Environmental, social, and governance scores lessen banking performance in both developed and developing countries
Chowdhury et al. [59]	Quantitative	Measured and compared efficiency between Islamic and conventional banks		DEA, Malmquist, Annual reports	Islamic banks exhibited better comparably in efficiency than conventional. All commercial banks need to improve managerial efficiency
Moudud-Ul-Huq [62]	Quantitative	Investigated the relationship between risk-taking behaviour and banks’ competition performance	competition-stability theory, quiet life hypothesis. Structure-conduct-performance	GMM, panel data	Bank size heterogeneously affects bank performance and risk-taking behaviour in emerging countries, and competition substantially affects bank performance
Santana [57]	Quantitative	Investigated the effects of banking crises and financial liberalisation on the relationship between financial development and economic growth		GMM, Dynamic panel data	Financial liberalisation did not show a positive relationship between financial development and economic growth due to the emergence and recurrence of banking crises
Zeqiraj et al. [61]	Quantitative	Investigated the dynamic impact of banking sector performance on economic growth		GMM, Dynamic panel data	The banking sector showed a significant positive effect on economic growth. It implied that banking efficiency is one of the key determinants of overall economic growth
Adesina [37]	Quantitative	Examined the effect of human capital efficiency on the link between diversification and bank performance	Intermediation	SFA, Tobit regression	Higher diversification reduces bank performance in three ways; cost efficiency, profitability and financial stability. Hence, higher levels of human capital efficiency were positively relating to bank performance
Bose et al. [38]	Quantitative	Investigated the effect of green banking to improve banks’ financial performance		Regression (OLS), panel data, annual reports	A positive relationship was found between green banking and banks’ financial performance, while cost-efficiency moderated this relationship. However, banks’ political connection negatively driven this relationship
Bhattacharyya et al. [39]	Quantitative	Investigated the relationship of CSR expenditures and financial inclusion on banking performance	Freeman’s stakeholder theory,	GMM, panel data	In terms of accounting measurement, CSR expenditure and degree of financial inclusion were not linked to banks’ financial performance, while a negative link was found in terms of the stock market return
Chen and Lu [43]	Quantitative	Examine the impact of the regional disparities in the cost and profit efficiency	Intermediation	SFA, Annual reports	Banking efficiency had a positive correlation to per capita GDP while negatively related to the urban population ratio
Chowdhury and Haron [5]	Quantitative	Measured efficiency of SEA Islamic banks	Intermediation	DEA, Malmquist, Annual reports	Islamic banks have improved inefficiency in the region
Gaies and Nabi [69]	Quantitative	Examined the interaction between FDI and external debt	The overlapping generation growth model	GMM, panel data	Banks posited an effect on economic growth. External debt financial enhanced vulnerability to a bank operation that generates a recessionary effect on economic growth
Haini [67]	Quantitative	Investigated the nonlinear impact of banking sector development on economic growth		GMM, dynamic panel data	A nonlinear relationship was found between banking sector development and economic growth. High levels of banking sector development push out the positive effect of private investment
Isnurhadi et al. [70]	Quantitative	Evaluated the relationship between bank capital, efficiency, and risk in Islamic banks		Pooled OLS and Random Effect (RE), panel data	Bank capital positively affects bank stability and negatively on credit risk and efficiency. hence, efficiency encouraged banks to lessen risk even when the capital was lower
Kchikeche and Khallouk [71]	Quantitative	Examined the causal link between banking financial development and economic growth		Vector autoregression framework	Bank-based financial development affected economic growth in both the short and long run
Lehmann-Hasemeyer and Wahl [66]	Quantitative	Revisited the effect of saving banks on economic development in Prussia		Regression (fixed effects)	A significant positive relationship was demonstrated between the establishment of saving banks and city growth in the nineteenth century
Li et al. [72]	Quantitative	Investigated the impact of credit risk shocks on the evolution of banking efficiency	Efficiency theory	DEA (bootstrap-DEA), annual report	The efficiency of both state-owned and joint-stock commercial banks was higher than urban/rural commercial banks during a credit risk shock
Rehman et al. [40]	Quantitative	Examined the effect of reformed bank sectors on the relationship between bank performance and board structure	Soft-budget constraint, intermediation	SFA, DEA, Regression, panel data	A negative link was found between board independence and banking efficiency; however, it became positive when banks were listed in the stock market. Larger banks are less efficient than smaller banks
Ryu and Yu [64]	Quantitative	Examined the nonlinear effect on bank performance due to changes in subordinated debt		Regression (FE, RE), panel data	Debt issuances adversely and significantly affected bank performance, and redemptions did not boost up this effect
Stewart and Chowdhury [73]	Quantitative	Investigate the effect of bank stability, liquidity and capital on the relationship between output growth and bank crises		GMM, Panel data	A stable banking sector reduces the negative effect of a crisis on GDP growth further provides economic resilience in developed and developing countries
Tam et al. [65]	Quantitative	Investigated the effect of independent directions on bank performance		Regression, panel data	Independent directors with a higher board hierarchy positively affect the bank performance and cost-efficiency, while the negative effect was estimated on the variability of performances
Yu et al. [41]	Quantitative	Assessed the dynamic performance of banks		DEA, panel data and annual reports	Inefficiency in the deposit process caused the overall inefficiency of the banks; thus, improvement in deposit efficiency was more important than lending efficiency

## Abbreviations

DEA: Data envelopment analysis
GMM: Generalized method of moments
WoS: Web of Science
CI: Collaboration index
CEO: Chief executive officer
CSR: Corporate social responsibility
